# Paediatric biepicondylar elbow fracture dislocation - a case report

**DOI:** 10.1186/1749-799X-5-75

**Published:** 2010-10-15

**Authors:** Mahendrakumar Meta, David Miller

**Affiliations:** 1 Orthopaedic Registrar , Department of Orthopaedics, Royal Brisbane & Women Hospital, Butterfield Street, Herston 4029, QLD Australia; 2 Orthopaedic RMO, Department of Orthopaedics, Royal Brisbane & Women Hospital, Butterfield Street, Herston 4029, QLD Australia

## Abstract

Paediatric elbow biepicondylar fracture dislocations are very rare injuries and have been only published in two independent case reviews. We report a case of 13 years old boy, who sustained this unusual injury after a fall on outstretched hand resulting in an unstable elbow fracture dislocation. Closed reduction was performed followed by delayed ORIF (Open Reduction and Internal Fixation) with K wires. Final follow-up at 14 weeks revealed a stable elbow and satisfactory function with full supination-pronation, range of motion from 0°-120° of flexion and normal muscle strength. This type of injury needs operative treatment and fixation to restore stability and return to normal or near normal elbow function. The method of fixation (screws or K wires) may depend on size and number of fracture fragments.

## Background

Upper extremity injuries are more common in children (65-75% of all fractures in children) as they tend to protect themselves with their outstretched arms when they fall [1]. Distal humerus fractures account for approximately 86% of all fractures around elbow. Whilst supracondylar fractures are the most common elbow injuries, they are closely followed by fractures of the lateral epicondyle and the medial epicondyle [1]. Medial epicondyle fractures are commonly associated with elbow dislocations. Lateral epicondyle fractures are rare. Isolated injuries are reported sparsely and mostly in textbooks like "Rockwood and Green's Fracture in Children" [1]. To our knowledge, biepicondylar fractures with an associated elbow dislocation are only reported twice in the literature [2,3].

Variations in appearance of different ossification centers around elbow add to the complexity and difficulty to diagnose and manage patients with this injury. The medial epicondyle begins to ossify at approximately 5 to 6 yrs of age with fusion occurring at approximately 15 yrs of age. The lateral epicondyle appears at about 10 yrs of age and is not always visible [1]. Therefore fractures may be easily overlooked due to its late and unusual pattern of ossification [3-5].

The mechanism of injury is complex and still remains to be resolved. Fifty percent of medial epicondyle fractures are associated with elbow dislocations with the ulnar collateral ligament causing an avulsion fracture. When a child falls on outstretched hand with elbow in full extension, the wrist and fingers are often hyperextended, resulting in tension forces on the medial epicondyle by the forearm flexors. In addition, normal valgus carrying angle accentuate these avulsion forces. The fracture fragment is incarcerated in the joint in 15-18% of patients [1]. In contrast, lateral epicondyle fracture can occur from a direct blow or avulsion forces from the extensor muscles [1]. A plausible explanation for the etiology of biepicondylar fractures could be the fact that during fall on outstretched hand, valgus forces at the elbow in combination with internal rotation of humerus over planted forearm and hand leads to traction and avulsion forces on both epicondyles [2].

Taylor et al [3] published the first case in a 9 yrs old girl following a fall whilst horse riding in 1997. The injury was treated with ORIF and K wires. The patient recovered to a painless, stable elbow with full range of motion at six months.

In 2008, Gani et al [2] reported a similar case of 13 yrs old girl with an unstable elbow joint following closed reduction. The author proceeded to ORIF of both epicondyles using screw fixation, which resulted in satisfactory elbow function at 5 months. Here the mechanism was a direct injury to the elbow caused by the fall of a heavy copper pot onto the involved elbow.

We report a case of biepicondylar elbow fracture dislocation in a 13- year-old boy, which was treated with ORIF and K wire fixation.

## Case Presentation

**Figure 1:**
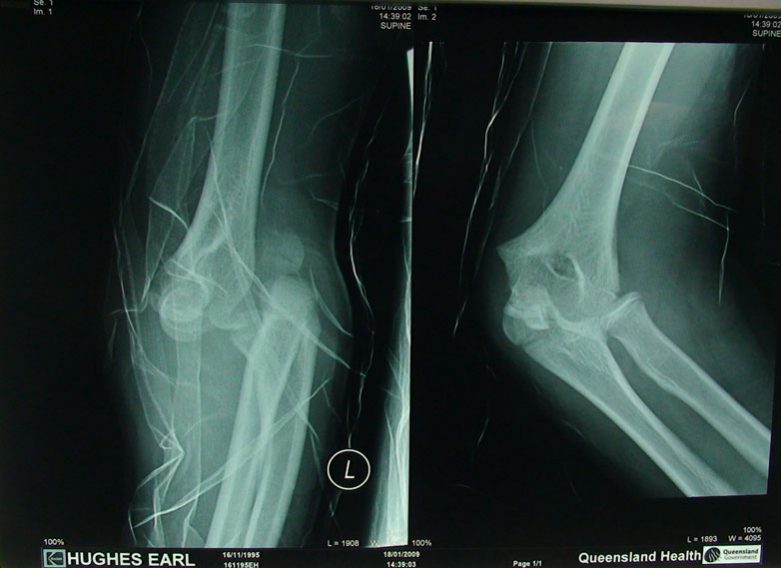
**Injury X-ray 1 (showing dislocated elbow with biepicondylar fractures)**.

**Figure 2:**
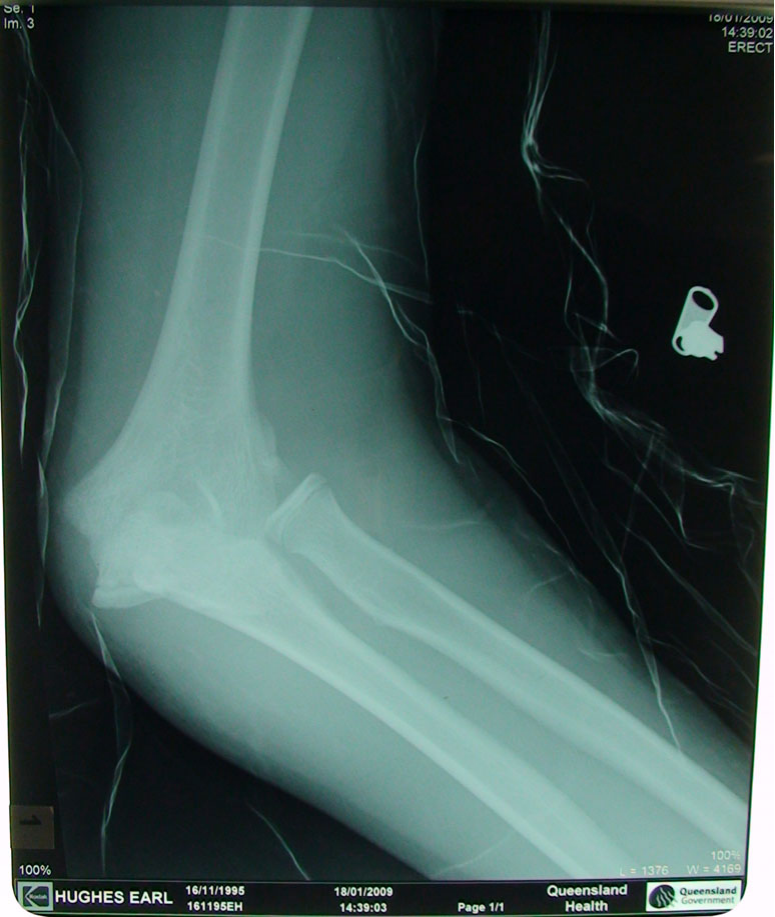
**Injury X-ray 2**.

**Figure 3:**
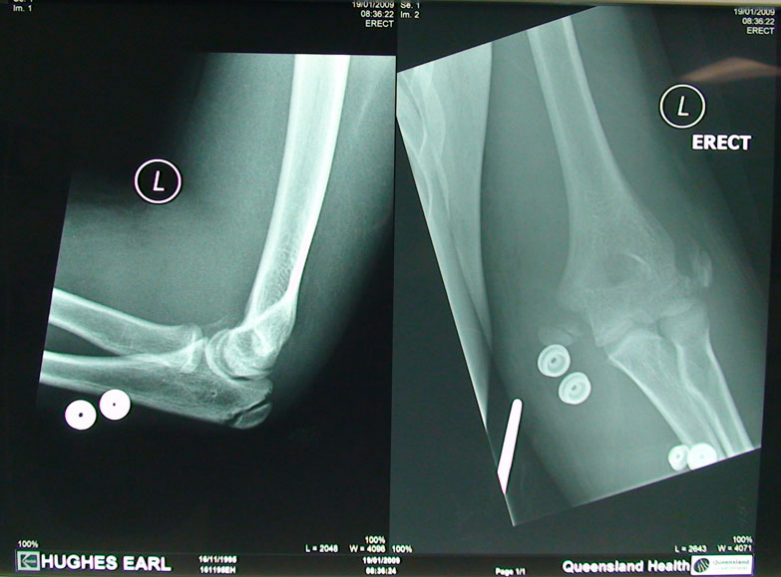
**Post reduction X-ray (showing reduced elbow with displaced biepicondylar fractures)**.

**Figure 4:**
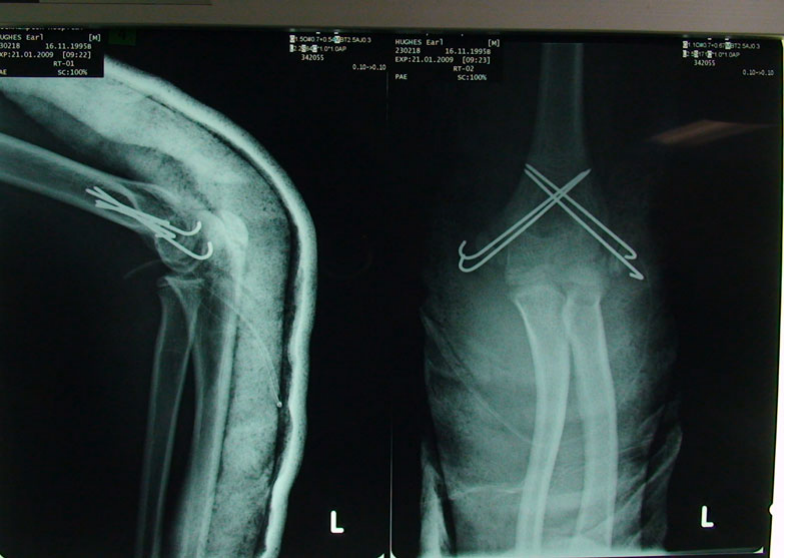
**Postoperative X-ray (showing fixation with K wires)**.

**Figure 5:**
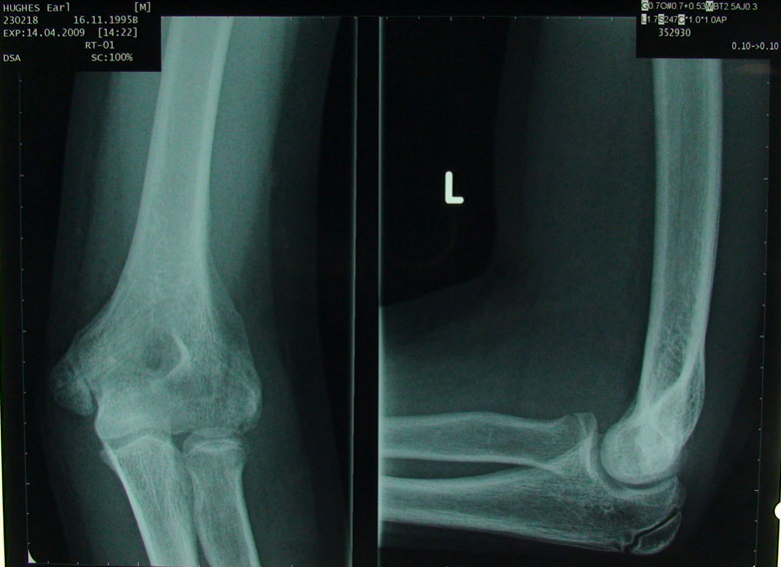
**Final follow-up X-ray (showing fully united medial and lateral epicondyles)**.

A 13 yrs old boy sustained a fall on his outstretched hand. He presented with a grossly swollen and deformed elbow. Radiographs demonstrated a posterolateral elbow dislocation with fractures of both the lateral and medial epicondyles (Figures 1 and 2 - showing three different views). The elbow dislocation was reduced and immobilized in the emergency department. Post-reduction radiographs showed a reduced elbow with displaced fractures of medial and lateral epicondyles (Figure 3- Post reduction radiographs demonstrating AP and Lateral views). However as the elbow remained clinically highly unstable and the fractures were still markedly displaced, operative intervention was deemed necessary. ORIF of both the medial and lateral epicondyles was performed using a separate medial and lateral approach. Due to the presence of fracture comminution and small sized fragments of both epicondyles, screw fixation was deferred. K wire fixation using two 1.6 mm wires for each the lateral and medial epicondyle was preferred. Post-operative radiographs showed satisfactory reduction and fixation (Figure 4- postoperative radiographs showing AP and lateral views after K wire fixation). Following six weeks of immobilization in a plaster of Paris, active elbow ROM (range of motion) was commenced by a physiotherapist. The patient received weekly physiotherapist treatment until week 14. K wires were removed at postoperative week eight. At the final follow-up 14 weeks postoperatively, satisfactory elbow function (0°-120° flexion, full supination and pronation, with normal strength and stable elbow) was observed. Radiographs demonstrated bony union and no evidence of myositis ossificans (Figure 5- Final follow up radiographs showing AP and lateral views of elbow with union of both epicondyles). Prophylactic treatment for myositis ossificans was not used.

## Conclusion

Biepicondylar elbow fracture dislocations are unstable injuries. Open reduction and internal fixation of these injuries is recommended to restore elbow stability and function.

## Consent

Written informed consent was obtained from the patient's parents for publication of this case report and any accompanying images. A copy of the written consent is available for review by the Editor-in-Chief of this journal.

## Competing interests

The authors declare that they have no competing interests.

## Authors' contributions

MM designed the study, collected data, wrote the manuscript and performed literature review. DM assisted in writing manuscript, literature review and obtained consent from parents. Both authors read and approved the final manuscript.
